# Sharing reports about domestic violence and abuse with general practitioners: a qualitative interview study

**DOI:** 10.1186/s12875-020-01171-4

**Published:** 2020-06-23

**Authors:** Katherine Pitt, Sandi Dheensa, Gene Feder, Emma Johnson, Mei-See Man, Jessica Roy, Emma Williamson, Eszter Szilassy

**Affiliations:** 1 Bristol Medical School (Population Health Sciences), University of Bristol, Canynge Hall, 39 Whatley Road, Bristol, BS8 2PS UK; 2 School for Policy Studies, University of Bristol, Bristol, UK

**Keywords:** Domestic violence and abuse (DVA), Intimate partner violence (IPV), Information-sharing, Multi-agency, General practice, Primary care, Police, Electronic medical records (EMR)

## Abstract

**Background:**

Domestic violence and abuse (DVA) is common and damaging to health. UK national guidance advocates a multi-agency response to DVA, and domestic homicide reviews consistently recommend improved information-sharing between agencies. Identification of patients experiencing DVA in general practice may come from external information shared with the practice, such as police incident reports and multi-agency risk assessment conference (MARAC) reports. The aim of this study was to explore the views of general practitioners (GPs) and the police about sharing reports about DVA with GPs.

**Methods:**

Qualitative semi-structured interviews were conducted with GPs, police staff and a partnership manager. Participants were located across England and Wales. Thematic analysis was undertaken.

**Results:**

Interviews were conducted with 23 GPs, six police staff and one former partnership manager. Experiences of information-sharing with GPs about DVA varied. Participants described the relevance and value of external reports to GPs to help address the health consequences of DVA and safeguard patients. They balanced competing priorities when managing this information in the electronic medical record, namely visibility to GPs versus the risk of unintended disclosure to patients. GPs also spoke of the judgements they made about exploring DVA with patients based on external reports, which varied between abusive and non-abusive adults and children. Some felt constrained by short general practice consultations. Some police and GPs reflected on a loss of control when information about DVA was shared between agencies, and the risk of unintended consequences. Both police and GPs highlighted the importance of clear information and a shared understanding about responsibility for action.

**Conclusion:**

GPs regarded external reports about DVA as relevant to their role, but safely recording this information in the electronic medical record and using it to support patients required complex judgements. Both GPs and police staff emphasised the importance of clarity of information and responsibility for action when information was shared between agencies about patients affected by DVA.

## Background

Domestic violence and abuse (DVA) is a violation of human rights that damages health, requiring a public health and clinical response. The UK Government defines DVA as:

*any incident of controlling, coercive or threatening behaviour, violence or abuse between those aged 16 or over who are or have been intimate partners or family members, regardless of their gender or sexuality* [1].

The 2018 Crime Survey for England and Wales found that an estimated 7.9% of women (1.3 million) and 4.2% of men (695,000) experienced DVA in the previous year [2]. Evidence suggests that the prevalence among patients accessing health services is even higher. A cross-sectional survey in primary care waiting rooms found that 17% of women had experienced past year and 41% lifetime physical violence from a current/ former partner [3]. However, DVA remains under reported in general practice [3, 4].

DVA is associated with substantial morbidity and mortality [2, 5, 6]. Women are disproportionally affected as victims [2]. At the same time, male victims may experience additional barriers to accessing help [7]. The physical and mental health burden affecting victims of DVA is well evidenced [5, 6]. Furthermore, exposure to DVA is a form of child maltreatment [8]. Children affected by DVA are at higher risk of growth, developmental, and behavioural problems [9] and other forms of child maltreatment [10].

DVA costs health an estimated £2.3 billion, police £1.3 billion and housing £550 million annually [11]. The National Institute for Health and Care Excellence (NICE) advocates a coordinated multi-agency response [12]. Domestic homicide reviews and child serious case reviews have consistently advocated improved information-sharing between agencies [8, 13, 14]. Professional guidance indicates that information-sharing between agencies may be justified (in specific circumstances without consent) to safeguard children or prevent serious harm to DVA victims [15, 16]. Multi-agency structures are established in the UK to facilitate information-sharing, namely multi-agency risk assessment conferences (MARAC) and multi-agency safeguarding hubs (MASHs) [17, 18].

General practitioners (GPs) have an important role in the multi-agency response to DVA, given its prevalence and health impact [3, 5, 6]. Some DVA cases, involving children or adults with care and support needs, are also relevant to doctors’ safeguarding responsibilities [19, 20]. Professional guidance supports doctors’ role in identifying and responding to DVA [19, 21]. Research indicates that both men and women feel it appropriate for doctors to ask about DVA [22, 23].

Previous research with GPs has identified concerns about documenting DVA in the patient medical record [24]. Studies show that GPs use various approaches to documenting DVA [25] and that DVA remains under-recorded in the medical record [3, 4]. National guidance exists about safely recording DVA in electronic medical records (EMRs) [26]. Patient online access to EMRs has led to further concerns about unintended breaches of confidentiality [27, 28].

A randomised controlled trial of a general practice-based training and advocacy intervention - Identification and Referral to Improve Safety (IRIS) – reported increased rates of identification and referral to DVA specialist support of women affected by DVA [29]. An adaptation of IRIS, IRIS+, has extended this intervention to also respond to children and men. The first stage of the IRIS+ feasibility study was conducted in four general practices in one region of England over eleven months between 2017 and 2018. Following IRIS+ training, electronic medical records (EMRs) in the study practices were searched to measure DVA identifications and documented GP responses during the study period.

An evaluation of the IRIS+ feasibility study findings is underway. Briefly, two-thirds of DVA identifications were from reports shared with general practice from another agency incorporated into the EMR, not consultations with a clinician. A large majority of third-party notifications were police DVA incident reports about families with children or MARAC reports about high-risk cases of DVA (Szilassy E, et al., Reaching everyone in general practice? Feasibility of an integrated domestic violence training and advocacy support intervention for primary care, unpublished). The importance of external reports as a source of DVA identifications in general practice was not anticipated in the IRIS+ study design. There was no documentation in the EMR to indicate how GPs were using or responding to this information. We explored attitudes towards these reports with four GPs who participated in IRIS+, and their views were variable. We wanted to explore professional attitudes towards sharing reports about DVA with GPs more closely. This led to an IRIS+ interview sub-study to explore GP and police staff views about DVA information sharing, reported in this paper.

A few studies explore information sharing between public sector organisations about DVA [30, 31]. However, little research explores professional attitudes towards sending reports about DVA to GPs. One study in Scotland found difficulties implementing information-sharing between the police and GPs in practice [32]. This study therefore addressed a gap in research. This study is timely, given national guidance advocating improved inter-agency information-sharing and a specific general practice response to DVA [12, 33].

## Methods

### Aims and objectives

The aim of this qualitative interview study was to explore the views of GPs and police staff about sharing information concerning DVA with general practice, with a view to informing the interpretation of the IRIS+ feasibility study findings and the possible reconfiguration of the IRIS+ intervention. The objectives of the study included exploring perceptions about the benefits and problems associated with sending reports about DVA to GPs, how they were used to inform patient care, and what was incorporated into the EMR. Police staff and MARAC chairs were interviewed, in addition to GPs, because most external reports about DVA identified in study practices were police incident reports or MARAC reports.

### Study setting

The research team was based at the University of Bristol and participants were recruited from across England and Wales. Recruitment was not limited to the area covered by the IRIS+ feasibility study because DVA information-sharing is relevant to professionals working in other localities.

### Study design

The study was a qualitative interview project. GPs were recruited by email advertisement to professional contacts, two regional clinical research networks and the IRIS network of practices [34]. The study was also promoted by two research posters at primary care conferences. Police staff were recruited from an advertisement on an electronic forum for a DVA charity. The sampling technique was predominantly convenience, based on response to the study invitation. All police staff who expressed interest were interviewed. Seven of the GPs were selected for known expertise in DVA or safeguarding. The final four GPs were purposively selected based on specific characteristics: prison service, region of the country, and male gender, with the aim of increasing the range of participants. In consequence, four participants who expressed interest towards the end of the study were not recruited.

Potential participants were sent a participant information sheet and consent form in advance. Informed written or recorded verbal consent was then taken for all interviews. Participants were offered a certificate and £15 shopping voucher as a gesture of gratitude for their time.

Interviews were conducted over a five-month period during 2018 by KP and SD. The topic guides were developed based on interviews with GPs involved in the IRIS+ feasibility study (see topic guides in additional files). The interviews were all semi-structured, which means that we used topic guides flexibly, probing relevant aspects of participants’ responses. KP, SD, and EJ met regularly to discuss all aspects of the study, including topic guides. The topic guides were adjusted during the data collection process in response to themes identified [35]. We added questions to the guide if we wanted more specific information about an issue. For example, as it emerged during the study that participants had different experiences of information-sharing, we added questions to the topic guide to explore participant perception of local information-sharing arrangements. The topic guide included the option of discussing a fictitious scenario in which a police report about an incident of DVA in a household with a child was shared with a GP practice. Interviews were audio-recorded and transcribed verbatim [36].

By the final interviews, the researchers felt that they had obtained sufficient depth and variation in the main concepts discussed, indicating saturation of the main themes [37]. GP and police transcripts were analysed as a single data set using inductive thematic analysis by three researchers (KP, SD and EJ) [38]. Each transcript was double coded using NVivo software version 11. During the analysis process, the team met to discuss how the codes identified could be organised into themes. KP led on reviewing the themes and designing a thematic structure. The final thematic structure was reviewed and agreed by SD and EJ.

### Ethics approval

The study was approved by the South West – Frenchay NHS Research Ethics committee (Reference number: 17/SW/0098).

## Results

Interview participants consisted of 23 GPs, six police staff and one former DVA partnership manager (who worked closely with one constabulary). Interviews were conducted by telephone or face-to-face (four, university premises or workplace). The duration of interviews varied, with an average of 35 min.

The demographic and professional characteristics of participants are summarised in Tables 1 and 2. GP participants worked in six different geographical regions of England and Wales. Police participants worked in five different geographical regions in England. GP participants were not from IRIS+ practice sites. Participants varied in their experience of information-sharing in relation to DVA: 47.8% of GPs interviewed had some experience of police reports and / or MARAC reports being shared with general practice. GPs described receiving external reports about DVA from other agencies, namely children’s social services as well as from Accident and Emergency departments. The police respondents had not shared reports directly with GPs; some described sharing information with MARACs or MASHs, which included representatives from the health sector. Participant attitudes did not vary consistently depending on demographic characteristic or professional experience.

**Table 1:** participant characteristics (GPs)

GP participants	Number
Age (years)	
30–39	9
40–49	6
50–59	4
60–69	4
Gender	
F	16
M	7
General practice role*	
GP Registrar	2
Locum GP	2
Salaried GP	4
GP Partner	9
Retired GP	2
Prison GP	2
GP (unspecified)	2
Specific DVA / safeguarding role*	
Current / former safeguarding lead	9
IRIS (DVA) trainer	4

* Some participants had more than one professional role

**Table 2:** participant characteristics (Police)

GP participants	Number (%)
Age (years)	
30–39	1
40–49	3
50–59	3
Gender	
F	1
M	6
Police role^*^	
Detective inspector	3
Sergeant	1
Police constable	1
Advanced practitioner	1
Former partnership manager	1
Specific DVA multi-agency role^*^	
MARAC chair	3

^*^Some participants had more than one professional role

Researchers identified four overarching themes during the process of analysis: [1] the relevance and value of external reports about DVA to GPs [2]; managing competing priorities in the EMR [3]; exploring DVA with patients - professional judgement and system constraints; and [4] the challenge of coordinating action between agencies.

### Theme 1: relevance and value of external reports about DVA to GPs

Notifications about DVA were considered valuable by GPs because they brought to light hidden issues of DVA and helped them to address the health consequences and safeguard children. These subthemes are explored below.

#### (1a) identify DVA that might otherwise remain hidden

Participants described some patients as reluctant to disclose DVA due to fear and mistrust of professionals. Some GPs described DVA as thereby under-identified, and external reports as key to addressing this.

*‘It’s really important for us to know this stuff. Actually, for a lot of these patients if it wasn’t for the police reports, we might not know it, we might not have asked about it, it might not have come up. It is valuable information.’* [GP 01]

#### (1b) address health consequences of DVA

GPs highlighted the adverse health consequences of DVA, and their role in addressing these.

*‘I think in terms of your question about what GPs can bring, I think we're absolutely central, because as we know with domestic abuse, the impact on your mental and physical health is massive. Therefore, it's really important that we know about it because it impacts on your overall wellbeing.*’ [GP 02]

GPs varied in how broadly they defined their role, from specifically addressing the health consequences to providing holistic support.

#### (1c) safeguard children

GPs regarded DVA to be a risk to children’s welfare and child safeguarding as a key responsibility. Participants discussed the importance of information-sharing between agencies and professionals in child protection. GPs valued external reports about DVA involving children, because they helped to contribute to a better understanding of potential risks to the child.

*‘I realise when we do our serious case reviews every time that there’s a child death and there seemed to have been domestic violence in the house and different agencies haven’t known what’s been happening.’* [GP 19]

### Theme 2: managing competing priorities in the EMR

While GPs regarded notifications about DVA as valuable, some raised dilemmas about how to record this information in patients’ EMR. These dilemmas are explored below.

#### (2a) balancing the visibility of information against the risk of unintended disclosure

GPs felt that DVA should be visible to GPs in the EMR, particularly when caring for victims and children. However, the visibility of information about DVA was balanced against the risk of unintended disclosure to patients, such as perpetrators.

*‘…there’s huge concern about recording and how to record and when to record, and how to redact, and when it’s confidential.’* [GP 05]

#### (2b) ownership of the EMR and labelling

Participants described a tension between the patient’s ownership and access to the EMR, versus its role as a repository of information assisting the safeguarding responsibilities of GPs.

*‘…there’s also the issue of these are patients’ notes, normally when we code something, like diabetes or something, we’d be having a discussion with the patient. Obviously, this is a bit sensitive, and I think, “Well, you don’t want to be paternalistic.” I’m still a bit trying to figure out what the best way is.’* [GP 12]

Some GPs were concerned about assigning labels within the EMR. A few participants felt that DVA could be regarded as stigmatising by patients (if they accessed the EMR). They also described the complexity of DVA and the risk of making assumptions based on information in external reports.

*‘…we don’t always know the full extent of the story. Perhaps it risks putting something, which can give somebody stigma, in their notes without having the full benefit of the facts.’* [GP 18]

### Theme 3: exploring DVA with patients - professional judgement and system constraints

GPs described the judgements they made about exploring DVA with patients, with additional complexities when information came from an external report. These judgements, and the constraints of the general practice consultation described by participants, are explored below.

#### (3a) navigating consultations with victims

Some participants reported that patient knowledge of, and consent for, information-sharing would influence how they responded. Without this, some GPs were concerned that enquiry based on third party reports may be intrusive or upset patients.

*‘If the police were to send the report to the GP, I think it’s important for those patients that they get their consent. I presume they are going to consent patients, to say, “Look, we are forwarding this information on to the GP, who may choose to contact you. Or you may choose to contact the GP directly.” I think if that conversation happens then that’s very good.’* [GP 09]

Participants described variation in patients’ recognition of abuse and readiness for support. GPs explained that the patients would often have a different agenda when they consulted, such as addressing a physical health complaint. Given this, some GPs favoured an incremental or indirect approach to asking about DVA, providing patients with the space to disclose DVA themselves. GPs talked of the value of building a trusting relationship with patients by supporting them with medical problems more specific to the GP role.

*‘There is something about […] relationships. I think we have a role in being able to address their problems, so actually it's making sure you've got effective contraception. You're really worried about your physical health. Yes, well, let's deal with that, if that's one less worry for you. It's not always about talking about domestic abuse all the time or abuse in general. It's about holistic care, isn't it?’* [GP 02]

Some participants reflected on the autonomy of adult victims of DVA. In contrast, others described the vulnerability of victims of DVA, and how coercion limited their freedom and capacity to make decisions. This tension could lead to uncertainty for professionals supporting victims of DVA about how to help, particularly when formal safeguarding procedures did not apply.

‘…*if there are kids or vulnerable adults, that’s fine, we know what we’re doing. It’s that grey area where survivors are falling into… people assume because safeguarding has been affected, that somehow the associated issues, be it the sexual violence or domestic abuse, substance misuse, have also been addressed and that’s absolutely not the case.* [Police 02]’

#### (3b) responding to perpetrators: risk and boundaries to the GP role

GP participants expressed concerns about causing an escalation of violence if they attempted to discuss perpetration. Some GP and police participants feared undermining the doctor-patient relationship.

*‘…actually GPs are about care, aren’t they? GPs are about providing care for someone who is in need of physical and sometimes mental health from a medical professional. If a perpetrator rocks up, who is perfectly entitled to his or her medical care, you run the risk of changing the perspective of that GP to the point where their decision making is affected because of that, and they no longer are giving perhaps the impartial service that they might have done.’* [Police 05]

Some participants described the GP role as supporting perpetrators with health problems that might exacerbate DVA, namely poor mental health and substance misuse.

#### (3c) constraints of the GP consultation with children

The constraints of the GP consultation were raised, particularly in relation to speaking to young children identified in external reports. Some participants highlighted the importance of hearing the perspective of children in households affected by DVA.

*‘…the reality is that we probably don’t ask the children enough and don’t hear their voice enough.’* [GP 01]

However, many participants felt that speaking to young children required sensitivity, which depended on time and rapport, whilst consultations were described as time-limited and mostly taken up dealing with unrelated problems. Participants also discussed the complexity of managing a consultation with a child brought in by their parent / carer, and difficulty seeing the child alone.

*‘Whereas in a paediatric setting or with specialists or safeguarding specialists often the infrastructure is there to allow them to see the children individually, to ask them questions and to build that rapport, but we just don’t get that opportunity really.*’ [GP 21]

### Theme 4: challenge of coordinating action between agencies

While GP participants acknowledged the value of external reports about DVA, both GP and police participants described difficulty coordinating action across organisational boundaries. These difficulties, pertaining to losing control when information is shared and clarity about responsibility for action, are explored below.

#### (4a) losing control when information is shared with another agency

A few participants feared that sharing information with external agencies or other practitioners could have uncertain consequences, particularly if the recipient’s confidence in responding to DVA was unknown.

*‘I’m not confident that if we shared the information with the GP, what the GP would actually do with it. That would range from nothing to potentially inappropriately sharing information’* [Police 03]

#### (4b) clarity of information and responsibility for action

Some police and GP participants argued that sending information about DVA to GPs could be conflated with inappropriately transferring responsibility for action.

*‘It would unfair to just say, “Well, we’ve sent it to you, your problem.”’* [Police 01]

Participants advocated thresholds to govern when information about DVA was sent to GPs, describing the high workload and limited capacity in primary care. They discussed the importance of a shared understanding between agencies about expectations when information about DVA was shared.

*‘I think we owe it to them that if we burden them with information around domestic abuse it’s only fair that we let them know what our expectations are around that.*’ [Police 05]

GPs too highlighted the importance of the clarity and content of the report itself.

*‘What we need is a way of highlighting domestic violence saying “This is what has been done, this is what is going to be done, this is what we need you to do as a GP”.’* [GP 21]

## Discussion

### Study findings

This study explored the views of GPs and the police about sharing reports about DVA with GPs. The GPs interviewed regarded external reports about DVA as relevant to their role. Participants perceived GPs as a potential source of support for people affected by DVA. However, there were barriers to directly addressing DVA in the GP consultation, particularly based on information from an external report. Patient knowledge of and consent to information-sharing was an important determinant influencing the response of GPs. Some participants were concerned about addressing perpetration in general practice, due to fears about undermining the doctor-patient relationship and causing an escalation of violence. Speaking to children about DVA was particularly challenging in time-limited consultations. Despite the difficulties with addressing DVA directly, GPs argued that information about DVA informed the care that they provided to patients. Recording this information within the EMR required GPs to balance visibility to clinicians against the risk of unintended disclosure to patients. Both GPs and police staff described the practical challenges of inter-agency information-sharing, and the importance of guidance for the recipient and clarity about responsibilities. Participant experiences of information-sharing varied. However, the variation in attitudes did not consistently correspond to experience of information-sharing or job role.

### Comparison to existing literature

GPs’ view of DVA as relevant to health care professionals reflects professional guidance [19, 21]. A previous study about DVA police reports sent to GPs in Scotland found similar perceptions [32]. Although participants in this study described GPs as a potential source of ongoing support, research with survivors indicates mixed experiences of gaining support from GPs, with some survivors concerned about GPs’ skill or experience asking about and responding to DVA [39, 40]. This echoes concern by participants in this study about information-sharing if the recipient’s competence in responding to DVA is unknown.

Concerns about discussing DVA mirror previous studies (not based on external reports), include time constraints, perceived patient reluctance to discuss DVA and stigma, and fear of making things worse [41–44]. GPs in this and previous studies discussed the role of trust in enabling discussions about DVA [41, 45]. In this study, GPs discussed the additional complexity of exploring DVA based on information in an external report. Interestingly, GPs in this study and in previous studies described strategies to frame inquiry that were often indirect and incremental [41, 45].

As with the current study, fears about discussing perpetration have been raised in previous research with GPs [46, 47]. Evidence indicates limited engagement by other professions with DVA perpetrators, for example social care [48, 49]. Some GPs described the complex dynamics of DVA, which may be unclear in an external report. GPs in other research studies have found it difficult to differentiate between perpetrators and victims of DVA in practice [46].

GPs in this study consistently highlighted the implication of DVA for child safeguarding. This link was identified variably in previous interview studies with GPs [42, 50]. GPs highlighted the importance of inter-agency information-sharing to building a picture of a child’s welfare. This reflects national policy guidance and learning from child serious case reviews [51]. Some GPs emphasised the importance of understanding children’s own perspective, an approach that national guidance supports [51, 52]. GPs also described this as difficult, especially given the topic’s sensitivity and time constraints [50, 53].

GPs described making complex judgements about recording DVA reports in the EMR. As with previous research with GPs about recording DVA and potentially stigmatising information in the medical record, some were reticent to record third-party information in alleged perpetrators’ EMRs, citing risk of unintended disclosures and concerns about labelling [25, 54]. GPs discussed tensions between their and their patients’ ownership of the EMR, and heterogeneous approaches to recording sensitive information [25, 55].

Participants reflected national policy in advocating a multi-agency response to DVA [12]. However, police staff were concerned about sharing information with professionals outside their organisation. As with a previous study, they were unsure how GPs would respond to reports about DVA [32], which may reflect GPs’ limited participation in multi-agency DVA collaborations [24]. Participants described the large workload and limited capacity in general practice. Previous research about police reports shared with children’s social services also described the workload implication [49]. Participants specified the need for clear expectations, when information was shared between agencies, reflecting previous research about DVA partnerships [48, 56].

### Implications for training and professional practice

General practice DVA training (IRIS) currently focuses on direct disclosures of DVA to a clinician. Since GPs also receive information from other agencies, they may benefit from advice about how to manage this information. This has been incorporated into GP training in the next stage of the IRIS+ feasibility study. Any guidance will need to reflect local multi-agency arrangements. We are also contributing to the development of guidelines about recording DVA from external reports in patient facing EMRs that balances visibility to clinicians against the risk of unintended disclosures to patients.

### Strengths and limitations

This study adds to the existing literature about how GPs respond to families affected by DVA and manage the EMR. It also complements existing research into the factors influencing the success of information-sharing and multi-agency working in the response to DVA and child safeguarding. The study benefited from having the perspectives of both GPs and police staff, and GPs with expertise in safeguarding and DVA and GPs without a specific role in this area. Participants were based in different regions of England and Wales and described different experiences of information-sharing.

The study had several limitations. Qualitative research does not aim to be generalisable or representative, so the perspectives of the participants cannot be presented as representative of GPs or the police more generally. The case scenario involved a child aged six, which may have shaped respondents’ comments about speaking to children. The interview study did not include other professional groups pertinent to the multi-agency response to DVA, such as social workers and health representatives on multi-agency safeguarding forums. Future studies should aim to explore the views of other professional agencies about DVA information-sharing. The police staff themselves had not shared reports directly with GPs (although some discussed sharing information with health representations on multi-agency forums). Some GPs had not received MARAC reports or police incident reports. While this means that their views were based on hypothetical scenarios, they were still able to share valuable insight into what the issues might be around information-sharing with GPs, based on, for example, their previous experience working with other services and agencies. Research is also needed to explore the attitude of people affected by DVA to information-sharing between agencies.

## Conclusions

GPs participants felt that notification about DVA from third parties helped them to treat the health impacts of DVA and safeguard vulnerable patients. However, incorporating this information into the EMR and using it to inform patient care in the consultation required careful professional judgement. Both police staff and GPs described the importance of clarity about expectations and responsibility for action when information about DVA was shared between agencies. GPs should be supported by colleagues with expertise in DVA and safeguarding in responding to this information. Training and guidance for GPs about DVA should explicitly address the challenge of recording and responding to information received from other agencies.

## Supplementary information

**Additional file 1.** Interview topic guide (GPs).

## Data Availability

Anonymised transcript data will be stored on the University of Bristol’s Research Data Service repository. Bona fide researchers will be able to access this data subject to a data access agreement and following approval from the University of Bristol Data Access Committee.

## Abbreviations

DVA: domestic violence and abuse
EMR: electronic medical record
GPs: general practitioners
IRIS: Identification and Referral to Improve Safety
IRIS+: Enhanced Identification and Referral to Improve Safety
MARAC: multi-agency risk assessment conference
MASH: multi-agency safeguarding hub
NICE: National Institute for Health and Social Care Excellence
