# Supplementary material for: Sharing reports about domestic violence and abuse with general practitioners: a qualitative interview study
Source: BMC Fam Pract. 2020 Jun 23;21:117. doi: 10.1186/s12875-020-01171-4 (PMC7313185; doi:10.1186/s12875-020-01171-4)
Supplement: Supplementary file 1 — Additional file 1. Interview topic guide (GPs). [file 12875_2020_1171_MOESM1_ESM.docx]

Additional files:

Interview topic guide (GPs)

| Table 1: topic guide for interviews with GPs |
| --- |
| 1. Tell me about the population you serve as a general practitioner? 2. How visible is DVA to you as a GP among the patients that you serve? 3. What information do you receive from other agencies about domestic abuse? 4. Optional case study:   *A police report is sent to your practice about a domestic incident. Three people are identified – a man, woman, and their 6-year-old child. All family members are registered at your practice. The woman alleged that the man hit her.*  What would you do if you received this police report? What would be recorded in the patient medical record? How would you approach the consultation if the woman, man or child subsequently attended an appointment? What variables might influence this?   1. How do you think patients feel about information being shared with GPs about DVA? 2. What is your experience of supporting patients affected by DVA in general practice? 3. How useful do you find external reports about DVA, in caring for your patients as a GP? 4. Why do you think this information is sent to GPs? 5. How should we be asking GPs to respond to external reports about DVA? |

Interview topic guide (police)

| Table 2: topic guide for interviews with police |
| --- |
| 1. Tell me about your professional role in responding to DVA? 2. Optional case study:   *The police are called to a domestic incident. A woman alleges that her male partner hit her. They have a 6-year-old child, who lives with them.*  Please describe how the police would respond to this incident, including risk assessment, support for the family and subsequent decisions about information-sharing with other agencies.   1. What information does the police force in your area share with health and specifically general practice in relation to DVA? 2. What is your attitude towards sharing information about DVA with general practitioners? 3. What is your expectation if information about DVA is sent to general practitioners? |
